# Diagnostic performance of regadenoson stress CMR for detection of coronary artery disease

**DOI:** 10.1186/1532-429X-17-S1-P94

**Published:** 2015-02-03

**Authors:** Mita Patel, Victor Mor-Avi, Sandeep Nathan, Roberto Lang, Amit R Patel

**Affiliations:** Cardiology, University of Chicago, Chicago, IL USA

## Background

The diagnostic performance of cardiovascular magnetic resonance (CMR) utilizing adenosine for detection of myocardial ischemia is well established. However, adenosine requires continuous infusion, which is challenging in the CMR environment, and has frequent side effects. The diagnostic accuracy of stress CMR using regadenoson, a newer vasodilator agent that is administered as a single bolus injection and has fewer side effects, has not been well studied. The aims of this study were: (1) to determine the diagnostic accuracy of regadenoson stress CMR when compared to coronary angiography in patients with suspected coronary artery disease (CAD), and (2) to study outcomes in patients with negative stress CMR perfusion studies to confirm that there are not a significant number of "abnormal studies" that are undetected.

## Methods

We studied 126 patients with suspected CAD referred for regadenoson stress CMR (1.5 T scanner, Philips). Stress perfusion images were obtained during the first pass of Gadolinium contrast agent one minute after regadenoson (0.4mg IV bolus) injection, and followed by reversal with aminophylline (75-125 mg). Cine imaging was followed by rest perfusion and late gadolinium enhancement imaging 10-15 minutes later. Patients were divided into 2 groups according to probability of CAD. Group 1 included 38 patients, who underwent coronary angiography within 6 months of CMR (Table), and was used to achieve goal 1. Significant CAD was defined by >70% coronary stenosis. Group 2 included 88 patients with normal regadenoson CMR (defined by normal right and left ventricular systolic volumes and function, absence of both visible perfusion abnormalities and LGE) who were followed-up for 12 months for cardiac death, myocardial infarction (MI), and revascularization. This group was used to achieve goal 2.

## Results

In group 1, 29/38 (76%) patients had significant CAD, and 35/38 (92%) had visible stress-induced perfusion abnormalities on CMR. The diagnostic accuracy of regadenoson CMR was 89%, sensitivity 93%, specificity 89%, positive predictive value (PPV) 96%, and negative predictive value (NPV) 72%. In group 2, none of the 88 patients had events within the follow-up period.

## Conclusions

This study provides one of the first assessments of diagnostic performance of regadenoson stress CMR in clinical practice, which was similar to published data for adenosine stress CMR. Importantly, the one-year event rate in patients with normal stress exams was <1/88, i.e. <1.14%. With its known advantages over adenosine, regadenoson is an effective alternative for cardiac MRI vasodilator stress testing in both patients with high and low probability of CAD.

## Funding

N/A.

**Table 1:** Patient Characteristics

Men, n	15
Women, n	23
No Prior Coronary Disease, n	18
Prior PCI and/or CABG, n	20
Hypertension, n	34
Hyperlipidemia, n	35
Diabetes, n	12
Tobacco Use, n	13
Family History, n	7

